# Ultra-high-resolution UAV-imaging and supervised deep learning for accurate detection of *Alternaria solani* in potato fields

**DOI:** 10.3389/fpls.2024.1206998

**Published:** 2024-03-05

**Authors:** Jana Wieme, Sam Leroux, Simon R. Cool, Jonathan Van Beek, Jan G. Pieters, Wouter H. Maes

**Affiliations:** ^1^Department of Plants and Crops, Ghent University, Ghent, Belgium; ^2^Technology and Food Unit, Flanders Research Institute for Agriculture, Fisheries and Food (ILVO), Merelbeke, Belgium; ^3^Internet Technology and Data Science Lab (IDLAB), Department of Information Technology at Ghent University - Interuniversity Microelectronics Centre (IMEC), Ghent, Belgium

**Keywords:** *Alternaria solani*, potato fields, supervised deep learning, UAV, sub-mm resolution, modified RGB, field-level

## Abstract

*Alternaria solani* is the second most devastating foliar pathogen of potato crops worldwide, causing premature defoliation of the plants. This disease is currently prevented through the regular application of detrimental crop protection products and is guided by early warnings based on weather predictions and visual observations by farmers. To reduce the use of crop protection products, without additional production losses, it would be beneficial to be able to automatically detect *Alternaria solani* in potato fields. In recent years, the potential of deep learning in precision agriculture is receiving increasing research attention. Convolutional Neural Networks (CNNs) are currently the state of the art, but also come with challenges, especially regarding in-field robustness. This stems from the fact that they are often trained on datasets that are limited in size or have been recorded in controlled environments, not necessarily representative of real-world settings. We collected a dataset consisting of ultra-high-resolution modified RGB UAV-imagery of both symptomatic and non-symptomatic potato crops in the field during various years and disease stages to cover the great variability in agricultural data. We developed a convolutional neural network to perform in-field detection of *Alternaria*, defined as a binary classification problem. Our model achieves a similar accuracy as several state-of-the-art models for disease detection, but has a much lower inference time, which enhances its practical applicability. By using training data of three consecutive growing seasons (2019, 2020 and 2021) and test data of an independent fourth year (2022), an F1 score of 0.93 is achieved. Furthermore, we evaluate how different properties of the dataset such as its size and class imbalance impact the obtained accuracy.

## 1 Introduction

The global annual production of potato surpassed 375 million tons in 2021 (FAO, 2023), making it the third most important food crop in terms of global consumption. However, a multitude of stress factors make potato production highly variable and unpredictable, which could result in lower yield. The control of these stresses requires significant amounts of plant protection products at regular intervals, with high economic and environmental impact as a result Afzaal et al. (2021). The application of these products is usually uniformly throughout the complete field, regardless of any spatial variation of possible in-field infestations Afzaal et al. (2021).

*Alternaria solani* is the second most destructive foliar pathogen in potato production and therefore an important stress factor in potato cultivation. It causes early blight, leading to premature crop dieback and hence crop yield reduction. Currently, a preventive, uniform application with agrochemicals is used to avoid these *Alternaria* infections. The application schedule follows the treatment scheme for *Phytophtora infestans*, which is mostly guided by early warning based on local and visual observations in the area, as well as on weather forecast systems Van De Vijver et al. (2020); Meno et al. (2021); Chakraborty et al. (2022). However, public awareness about the impact of both the uniform and frequent application of protection products is increasing, creating an imminent demand for more efficient use. The European Commission also targets a reduced use of agrochemicals in their Farm to Fork strategy European commission (2022) to stimulate the development of new agricultural management practices. As application timing is crucial for controlling *Alternaria solani*, automatic detection of early symptoms will be beneficial to enhance early warning systems. If early symptoms can be identified and mapped, task maps can be created for variable spraying. Early warning systems and variable spraying can reduce the consumption of protection products while minimizing both production losses and the economic and environmental impact Van De Vijver et al. (2020); Afzaal et al. (2021); Anim-Ayeko et al. (2023).

Detection of disease symptoms using computer vision has been extensively studied, traditionally using classical image processing and machine learning and more recently also using deep learning algorithms Thakur et al. (2022); Kamilaris and Prenafeta-Boldú (2018). Deep learning surpasses traditional methods by automatically learning features from large datasets, instead of relying on manual feature engineering. It thus allows the variability in e.g., growth stages, shape, color, size, cultivar, growth and capture conditions to be captured Barbedo (2016).

Deep learning is also gaining attention in potato cultivation. In recent literature, a wide diversity of applications can be found, both in terms of the application itself and in terms of data collection and spectral characteristics. The automated detection of disease symptoms on leaves, plants or tubers, including blight detection, is a frequently recurring application in literature. Where Van De Vijver et al. (2020); Iqbal and Talukder (2020) and Hou et al. (2021) mainly used traditional machine learning techniques, CNNs are addressed in Sholihati et al. (2020); Tiwari et al. (2020); Afzaal et al. (2021); Johnson et al. (2021); Khalifa et al. (2021); Rashid et al. (2021); Chakraborty et al. (2022); Chen et al. (2018); Van De Vijver et al. (2022); Anim-Ayeko et al. (2023); Mzoughi and Yahiaoui (2023); Xia et al. (2023). Remarkably, all of these studies - except Van De Vijver et al. (2022) - focused on images of single leaves, presumably as a result of the publicly available PlantVillage dataset Hughes and Salathé (2015). This public dataset consists of leaves on a neutral background and is used in the majority of the studies. Only Afzaal et al. (2021); Johnson et al. (2021) and Xia et al. (2023) trained CNNs utilizing datasets focusing on a more complex field-level background, acquired using, respectively, digital hand-held cameras, smartphones, and a combination of smartphone and Internet data.

Despite this academic interest, the number of operational applications of deep learning in potato cultivation is still limited. Most of the studies are a proof of concept rather than an operational method directly applicable in practice Kamilaris and Prenafeta-Boldú (2018). Supervised models currently provide the best results in various sectors and are the standard in agriculture as well, but they come with a series of disadvantages that make real-time in-field disease detection difficult. The quality of supervised models heavily depends on the quantity and quality - and thus also the variability - of the dataset on which the model is trained. Quite often, datasets with insufficient size and variation are used Liu and Wang (2021). As Thakur et al. (2022) concluded in their review, most studies are performed on datasets in controlled conditions but fail generalize enough to be usable in the field. Gathering a qualitative dataset to develop a robust deep learning model is a relevant and challenging bottleneck that still hinders the effective dissemination of these models Barbedo (2018); Lu et al. (2021).

There are often practical reasons for this lack of application. First, the dataset needs to accurately reflect how the model will be used in practice. Since most models work on leaf-level images captured from a perpendicular angle with uniform backgrounds, applying them to field-level data becomes challenging Afonso et al. (2019). Abade et al. (2021) noticed that more than 65% of the reviewed studies were working in controlled environments [e.g. PlantVillage dataset Hughes and Salathé (2015)]. Hence, there is a need to move beyond these controlled environments, which indicates that data should be acquired in the field, without harming the crops.

In recent times, rapid and non-intrusive collection of field data has been facilitated through the use of smartphones Johnson et al. (2021); Xia et al. (2023). Yet, the downside is that this does not scale to larger fields. In addition, potato plants are sensitive to disturbances after crop closure, so tractor-mounted sensors are not always convenient either. Satellite data can provide a solution to easily monitor large fields without crop disturbance, but have limited resolution and often experience cloud inference. However, in early stages of infection, damage is often only visible as small spots on the leaves, and detection therefore requires ultra-high resolution data. Van De Vijver et al. (2020) concluded that, for the detection of *Alternaria* lesions, spatial context is essential and possibly more important than the combination of multiple wavebands in the visible and near-infrared (NIR) range Van De Vijver et al. (2022). In contrast to smartphones, tractor-mounted sensors, or satellites, Unmanned aerial vehicles (UAVs) provide a highly flexible means of scanning fields without disturbance, as they can be applied at the desired resolution and at the desired time, independent of the application of crop protection products. Such technology has only recently become available Maes and Steppe (2019).

Second, there is a need for sufficiently large datasets to cover the large variability (e.g., growth stages, cultivars, soil types) in agricultural data and the disease progression with deep learning Shorten and Khoshgoftaar (2019); Liu and Wang (2021). Thakur et al. (2022) provided an overview of frequently used publicly available datasets in the context of disease detection, including the type of image background. As mentioned before, PlantVillage [Hughes and Salathé (2015)] is the most widespread. Rashid et al. (2021) showed the limiting variation in PlantVillage for potato disease detection by testing a model, trained on PlantVillage, on their own Potato Leaf Dataset (PLD). Results showed that the overall accuracy was limited to 48.89%, compared to 86.38% in the inverse situation. This insufficient variation in the training set is also demonstrated by Mohanty et al. (2016): while reaching an overall accuracy up to 99.34% on the PlantVillage dataset [Hughes and Salathé (2015)], the accuracy reduced to 31.4% when testing on a dataset under different conditions. It can be concluded that no in-field dataset is readily available for early blight detection.

With limited publicly available datasets, most studies rely on a single dataset for both training and testing Lu et al. (2021). When the test set is extracted from the same database as the training set, even though no overlap exists, the high similarity between them can lead to decreased model accuracy when classifying entirely new data with more diverse characteristics Barbedo (2018). Lu et al. (2021) described this phenomenon as nonideal robustness, where it is assumed that training and test sets have the same distribution. However, this is not controllable in an in-field application caused by, for example, varying weather conditions or cultivars, and therefore is often associated with overfitting. Consequently, those models are often not robust enough to apply in different situations.

Finally, larger datasets from different fields imply more experimental and measurement efforts as well as an increase in labeling effort. Labeling is a time-consuming, tedious and laborious task. In some studies, models are trained to segment the images, requiring individual labeling for each lesion or even pixel in the image [Hou et al. (2021); Iqbal and Talukder (2020); Mzoughi and Yahiaoui (2023); Xia et al. (2023)]. Object detection requires slightly less labeling effort, although individual labeling for each lesion remains necessary [Johnson et al. (2021); Oishi et al. (2021); Van De Vijver et al. (2022)]. In classification, labeling involves assigning a class name to each input sample, a less specific but also less time-consuming task [Sholihati et al. (2020); Tiwari et al. (2020); Afzaal et al. (2021); Khalifa et al. (2021); Rashid et al. (2021); Chakraborty et al. (2022); Chen et al. (2018); Anim-Ayeko et al. (2023)]. A trade-off must therefore be made between the desired output detail and the labeling effort, with consideration for field-level implementation.

In previous work, we already demonstrated the feasibility of using deep learning models for *Alternaria* detection with modified ultra-high-resolution UAV data Wieme et al. (2022); Van De Vijver et al. (2022). In this study, we include a much larger dataset and propose a different, faster model dubbed *AlternarAI*. We compare its results on the collected dataset with those achieved by state-of-the-art CNNs and address the following research questions:

1. How does the AlternarAI model generalize over different years?
2. What is the impact of dataset characteristics such as size and class imbalance on the results of the AlternarAI model?
3. How does the model perform when used at field-level on a completely independent dataset?

## 2 Materials and methods

### 2.1 Field trial and data acquisition

Data were collected from both symptomatic and non-symptomatic plants during four consecutive growing seasons, 2019, 2020, 2021 and 2022, on 40×20 m experimental fields with sandy loam soil in Lemberge (Merelbeke), Belgium. The experimental field was yearly located within the same larger agricultural parcel (50.986544°N, 3.774066°E), although year by year on a different section of it. The field trial is conducted in analogy to the method described by Van De Vijver et al. (2020, 2022). Potato seedlings of different cultivars in the different growing seasons (**Table 1**) were planted with a distance of 0.4 m between tubers in the row, and 0.75 m between rows. To obtain a dataset with sufficient variation, and hence construct a robust model, different cultivars were used during different years. The choice was made by a plant pathologist, focusing on varieties with medium to high susceptibility to *Alternaria solani*, namely Spunta Nassar and Adss (2016), Bintje Van De Vijver et al. (2020) and Fontane Duarte et al. (2014).

**Table 1:** Overview parameters field trial.

(A): Field experiment				
Year	Variety	Inoculation date	Concentration spores (per ml)	Number (size) of plots
2019	Spunta	07/30/2019	3×10^3^	4 (3×2m)
2020	Bintje	07/06/2020	2×10^3^ 0.5×10^3^	4 (4×3m) 4 (4×3m)
2021	Bintje	06/28/2021	1.7×10^3^	4 (4×3m)
2022	Fontane	06/29/2022	1×10^2^	4 (4×3m)
(B): Flight measurements				
Year	Flight date	Days after inoculation (DAI)	Weather conditions	Total number of images (Other + Alternaria plots)
2019	08/02/2019	3	Cloudy, rain showers	564 (393 + 171)
	08/05/2019	6	Partly cloudy, warm	262 (168 + 94)
	08/08/2019	9	Cloudy, warm	670 (473 + 197)
2020	07/11/2020	5	Sunny, variable cloudy	732 (458 + 274)
	07/13/2020	7	Sunny, warm	964 (567 + 397)
	07/15/2020	9	Cloudy, light rain showers	562 (335 + 227)
2021	07/02/2021	4	Sunny, warm	484 (438 + 46)
	07/05/2021	7	Partly cloudy	506 (449 + 57)
	07/07/2021	9	Partly cloudy	493 (430 + 63)
	07/09/2021	11	Cloudy	266 (234 + 32)
2022	07/04/2022	5	Sunny, warm, dry	592 (545 + 47)
	07/06/2022	7	Sunny, warm, dry	619 (571 + 48)
	07/08/2022	9	Sunny, warm, dry	621 (564 + 57)
	07/12/2022	13	Sunny, warm, dry	621 (568 + 53)

Every year, four to eight plots with sizes between 3×2*m* and 4×3*m* were inoculated, after crop closure, with concentrations of *Alternaria solani* spores varying between 0.5×10^3^ and 2×10^3^ per ml, as shown in **Table 1**. The plots were randomly distributed across the field to randomize effects of various field characteristics. On the one hand, sufficient distance between the various plots was taken into account to avoid spreading infection. On the other hand, the plots were placed at a sufficient distance from the tractor tracks to avoid previous and further related damage. For the reference point of each plot, a row number was predetermined within the field, as well as a preliminary choice of distance from the edge of the field within that row. The exact placement within the defined row was adjusted based on visual evaluation of the crop, to limit damaged plants (e.g. due to presence of Colorado potato beetles) within the plot as much as possible.

**Figure 1:**
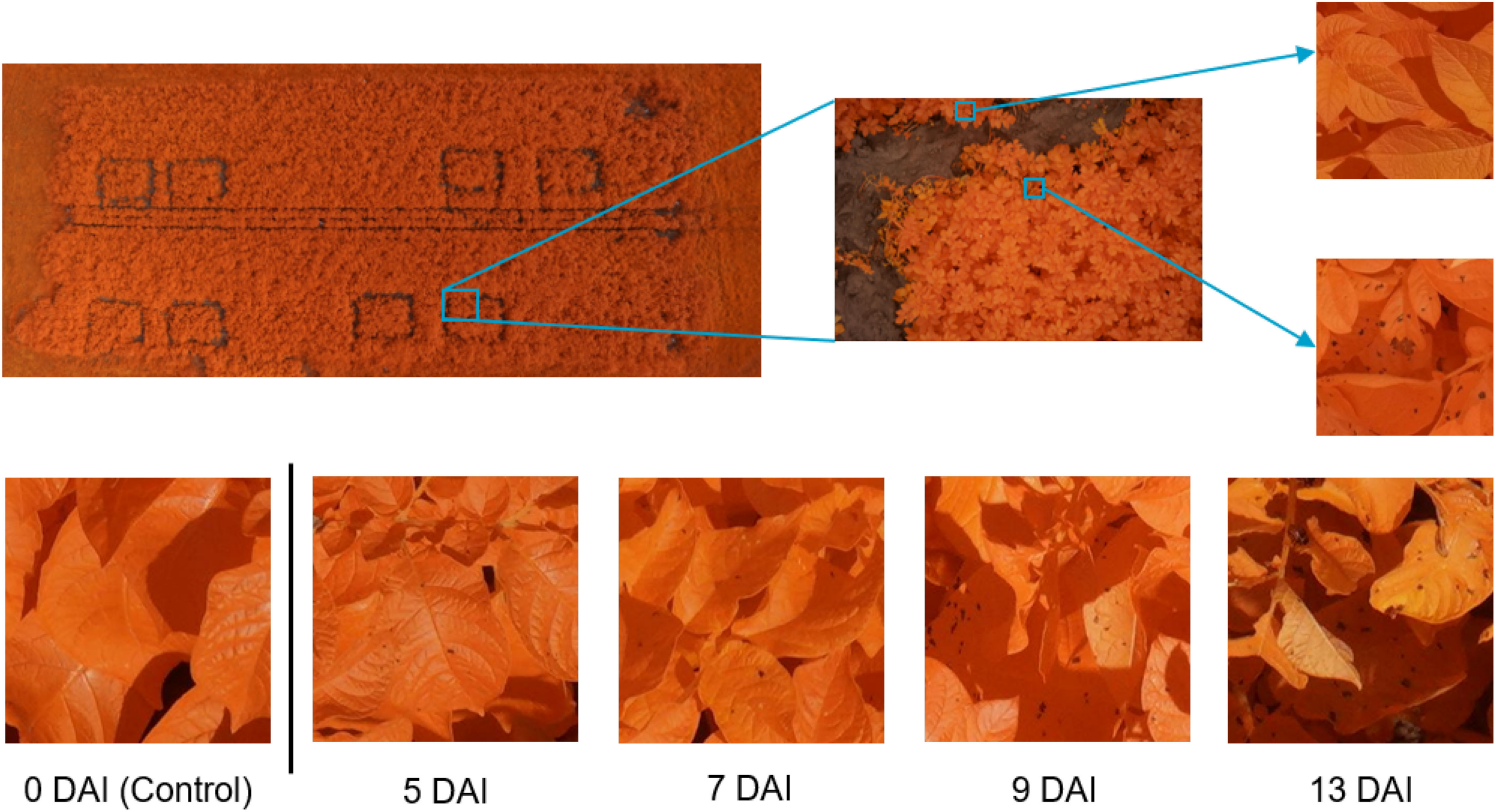
Dataset overview and examples. Top row: *left*: overview image of the field trial in July 2020 with 8 plots of 4 m × 3 m; *middle*: example of modified RGB image; *right*: *Alternaria* (bottom) and other patch (top). Bottom row: example patch per measurement day in 2022, showing the evolution of the *Alternaria* symptoms, starting from the day of the inoculation (control data) until 13 Days After Inoculation (DAI).

The inoculum was obtained by growing local field isolates under near-UV light on V8 agar plates. In the evening of the inoculation day, 14 days after the last field treatment, all plants in the predefined plots were homogeneously sprayed with 400 mL of inoculum using a backpack sprayer (Birchmeier, Stetten, CHE). To obtain leaf wetness for a sufficient long period and promote infection, the plants were covered at night with a tent structure of plastic tubes and clear film. The morning after inoculation, the uncovered part of the field received an extra treatment against *Alternaria solani*. An overview of the varieties, inoculation dates, concentration of spores and number of plots per year is given in **Table 1A**. In **Figure 1**, on the left top row, an overview image (modified RGB) of the field trial of 2020 is shown. In 2019 and 2020, in addition to the infected plots, four control plots were selected within the field as baseline for the visual monitoring. These were not sprayed with *Alternaria* spores or covered with a tent construction, and treated against *Alternaria* the morning after inoculation like the rest of the field. These control plots were defined analogously, taking into account sufficient distance from the infected plots and tractor tracks, and spread over the entire field. In 2021 and 2022 it was decided not to lay out these control plots explicitly anymore, as the remainder of the field was treated and monitored in the same way.

Starting the day after inoculation, plots were monitored visually on a daily basis by a trained plant pathologist. At the time of the first small symptoms, at the underside of the lower leaves Van De Vijver et al. (2020), although not yet visible from the top viewpoint in the visual spectrum, the first flight was performed. Based on this visual ground truthing, subsequent flights were scheduled at 2-3 days intervals, depending on weather conditions, resulting in measurements of twelve different days (three or four per growing season, details in **Table 1**). A DJI M600 PRO (DJI, Shenzhen, CHN) unmanned aerial vehicle (UAV) was equipped with a modified Sony Alpha 7III camera (42.4 MP) (Sony, Tokyo, JPN) with a 135 mm lens, (Batis 135 mm f2.8, Carl Zeiss AG, Oberkochen, DEU) for data acquisition. To allow the red channel of the camera to capture photons in the NIR range as well, the near-infrared (NIR) blocking filter in the RGB version of the camera was replaced by LifePixel (LifePixel, Seattle, USA) Maes and Steppe (2019). The choice to use a modified RGB camera, thus sensitive in the NIR, was based on previous work by Van De Vijver et al. (2020). This work showed that, although lesions were noticeable in the visual spectral region, the distinction between the healthy and infected pixels was more prominent and showed earlier in the near-infrared spectrum due to the disrupted leaf structure. To stabilize the camera and keep it in nadir-looking position, a gimbal, type DJI Ronin-MX (DJI, Shenzhen, CHN) was used. The flights took place at an altitude of 10 m to obtain the ultra-high resolution [0.3 mm/pixel Van De Vijver et al. (2022)], without UAV-downwash affecting the crops. The camera acquired top-view images of the crops with a field of view of about 2.5 m × 1.5 m, as shown in the middle of the top row of **Figure 1**. In 2019, 2020 and 2021, coordinates were assigned to all images based on the UAV logs. In 2022, the UAV was equipped with an extra RTK (Real Time Kinematic) module immediately adding the coordinates to the metadata of the images. More details about the flight measurements are given in **Table 1B**. In total, 7956 images were captured over the four consecutive years.

### 2.2 *Alternaria* Dataset

**Figure 2:**
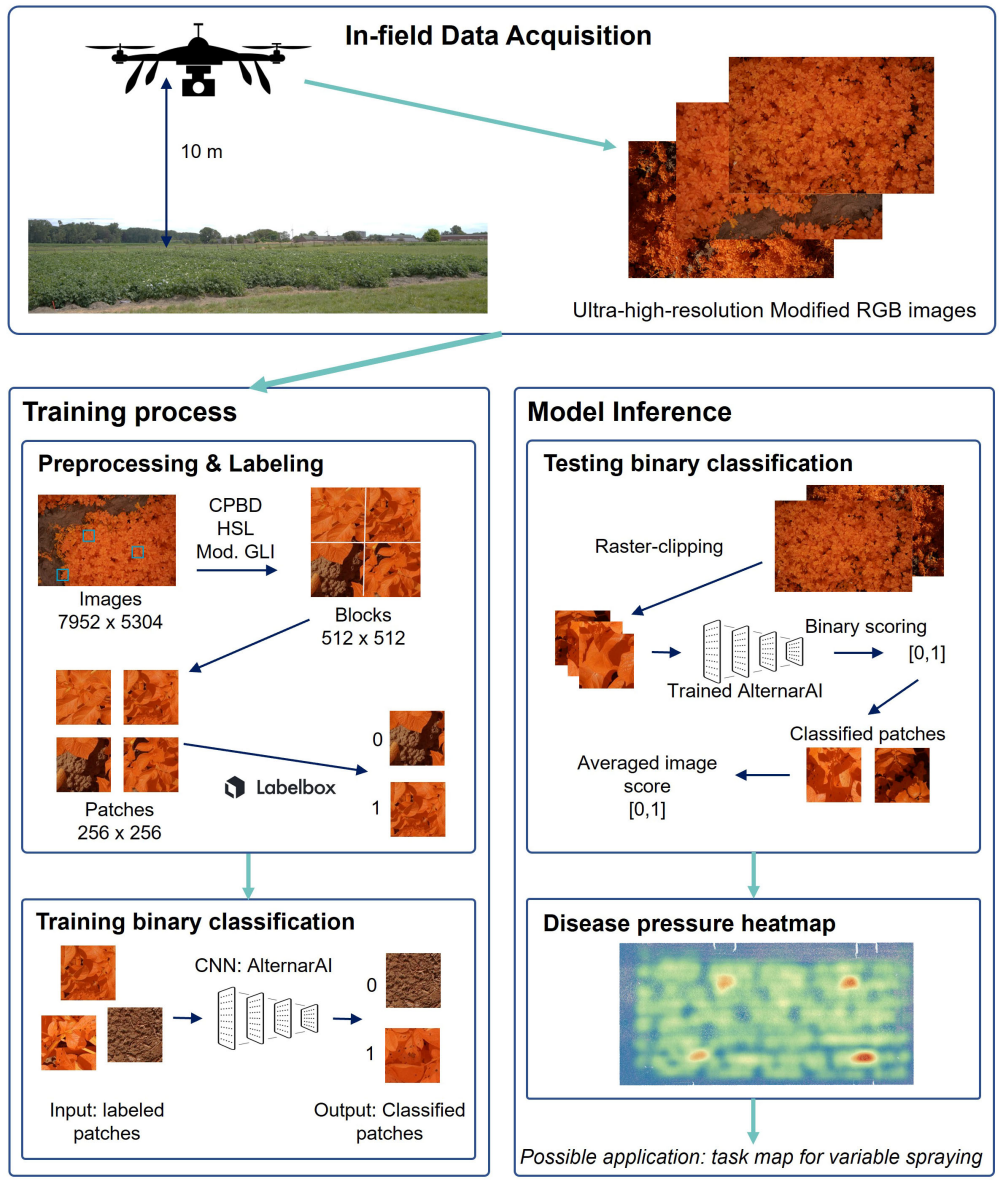
Visualization of the entire workflow. First step is in-field data acquisition. Left: training process; Right: model inference toward application.

The total dataset comprises data from fourteen different flights. As the goal is to create a supervised classification model with a binary output (*Alternaria* or not), binary labeled data is needed. The ultra-high-resolution images were first divided into non-overlapping patches of 256×256 pixels to serve as input for the model. Therefore, a preprocessing pipeline was created to select a subset of image patches in order to obtain a subset with sufficient variation. Subsequently, the selected subset was labeled, from which the final dataset was then created for further processing. These two steps, and the resulting experimental dataset, are described in the following three subsections and shown in the left half of **Figure 2**. The bottom row of **Figure 1** illustrates the evolution of the *Alternaria* symptoms in patches on the different measurement days in the campaign of 2022.

#### 2.2.1 Preprocessing pipeline

The first step in the preprocessing pipeline consisted of making a random selection from the images per measurement day. In this selection, attention was paid to the ratio of number of images from *Alternaria* plots to the total number of selected images per day to avoid an unbalance between both categories. In addition, an equal number of images from each *Alternaria* plot were selected per measurement day.

To perform this selection, all images were categorized based on their coordinates, compared to the coordinates of the vertices of the plots measured with an RTK-GPS (S10 GNSS Receiver) (Stonex, Milan, ITA), in QGIS QGIS.org (2020). For the data of 2019 and 2020, a ratio of 75/25% *Alternaria*/other was used, in 2021 50/50% and in 2022 62/38%, always with equal amounts of each measurement day. The reason for the slight oversampling of *Alternaria* plots is because *Alternaria* plots often still have non-symptomatic parts present. This resulted in 1525 selected images.

Next, four blocks of 512×512 pixels were selected from each selected image. To avoid the selection of irrelevant blocks, a three-step selection process was performed to evaluate the sharpness, illumination and presence of vegetation pixels, analogous as the flow described by Wieme et al. (2022). Each image (7952×5304 pixels) was split up into blocks of 512×512 pixels. The sharpness of each block was calculated using the perceptual-based no-reference objective image sharpness metric (CPBD) Bhor et al. (2013) and only blocks with sufficiently high CPBD-value (threshold value of 0.55) were processed further. Then, an illumination threshold was set to avoid lots of shadow areas, based on the lightness value in Hue, Saturation and Lightness (HSL). Last, to stimulate the selection of blocks containing plants, the green leaf index (GLI) Louhaichi et al. (2001) was adapted to the modified RGB images by swapping the green and red band in the Equation 1 and executed on all remaining blocks. The four blocks with the highest modified GLI-value, being at least 0.5, were selected for further processing. If fewer than four blocks met the selection criteria, fewer blocks of that image were included.


(1) $$\text{Modified GLI}=\frac{2R-G-B}{2R+G+B}$$

In the third step, the selected blocks of each image were raster-clipped into four patches of 256×256 pixels (shown in **Figure 1**), resulting in, in case all criteria were met, sixteen patches per selected image. As an addition to this preprocessing pipeline, to increase the variation and usability of the dataset, another 480 random patches were manually added, from the total set that had not been touched by the previously predetermined selection criteria. The complete preprocessing pipeline resulted in a selection of 22,722 patches for the labeling campaign.

#### 2.2.2 Labeling workflow

All 22,722 selected patches of 256 × 256 pixels were uploaded to the Labelbox platform Labelbox, Inc (2020). The patches were labeled into four different classes: “Alternaria”, “Healthy”, “Dubious” or “Background”. Afterwards, these four different classes were reduced to two categories for performing binary classification; “Alternaria” (category 1) or “Other” (category 0), covering both “Background” and “Healthy” patches.

Each patch undergoes labeling by three different annotators, resulting in three assigned labels. Only patches exhibiting sufficient consensus were considered, leading to the definition of four distinct situations. The initial scenario pertains to patches that received an identical label from all three annotators, achieving 100% consensus. The remaining three scenarios encompass patches with two instances of one label and one instance of a different label. In the first scenario, if a patch was labeled “Alternaria” twice and “Dubious” once, the consensus label assigned was “Alternaria”. In the second scenario, patches labeled twice as “Healthy” and once as “Dubious” or “Background” were categorized as “Healthy”. In the third scenario, a patch labeled twice as “Background” and once as “Healthy” was designated as “Healthy”. Patches not falling into these four scenarios were excluded from further consideration.

After processing the various labels and scores, a final dataset of 14,057 patches was extracted, consisting of 7909 patches of category “Other” and 6148 patches of category “Alternaria”, as shown in **Table 2**. From the 7909 patches of category 0, and 6148 patches of category 1, respectively 4225 and 4388 were labeled with 100% consensus.

**Table 2:** Size of labeled dataset by year and category.

Year	Other	Alternaria	Total
2019	1708	2795	**4503**
2020	2732	1739	**4471**
2021	1337	585	**1922**
2022	2130	1027	**3157**
**Total**	**7909**	**6148**	**14 057**

### 2.3 Supervised classification

We compared several models for the task of supervised *Alternaria* detection. All models were implemented using the Pytorch library Paszke et al. (2019) using Python 3.9 and trained using an NVIDIA GeForce RTX 3090 GPUs with CUDA 11 Nickolls et al. (2008).

#### 2.3.1 Experimental datasets

In all experiments, data augmentation in the form of random horizontal and vertical flipping of the training images was used to create additional variation in the dataset and to reduce the problem of overfitting Shorten and Khoshgoftaar (2019). Additionally, all data was normalized - per channel - by subtracting the average value and then dividing by the standard deviation, as determined in the specific data (sub)set. Different subsets of the data were created for the different experiments of Section 3. More details are provided in the respective subsections of Section 3.

#### 2.3.2 Model architectures

Over the last decade, various novel CNN architectures have been proposed, each with their own advantages and applicability scenarios Kamilaris and Prenafeta-Boldú (2018). In previous works on blight detection, different architectures have been used, such as VGG [Sholihati et al. (2020); Tiwari et al. (2020); Afzaal et al. (2021); Chakraborty et al. (2022); Anim-Ayeko et al. (2023), ResNet (Chakraborty et al. (2022); Anim-Ayeko et al. (2023), Inception (Tiwari et al. (2020)] and EfficientNet [Afzaal et al. (2021)]. Taking these into account, and to add more variation in older and newer models, as well as in size of the architecture, five state-of-the-art architectures, pretrained on ImageNet are compared: VGG11 Simonyan and Zisserman (2015), ResNet50 He et al. (2015), InceptionV3 Szegedy et al. (2015), DenseNet161 Huang et al. (2016) and EfficientNetV2 Tan and Le (2021), all available in the Pytorch library Paszke et al. (2019).

For each network, two different approaches of transfer learning are compared where either the full model is finetuned using our own dataset (deep transfer learning) or where all layers are kept fixed except for the last layer (shallow transfer learning). In the deep transfer learning approach, we trained the entire network by updating all layers, on our dataset with batch size 128. For all models except VGG11, we used 0.001 as initial learning rate in combination with Adam Kingma and Ba (2014) together with an L2 regularization of 0.0001. We trained for 100 epochs. This configuration of hyperparameters proved to function optimal for these models. For VGG11, we used an initial learning rate of 0.0001, as the model was - presumably due to the large amount of parameters - unable to deal with the smaller and unbalanced subdatasets in the other configuration. In the shallow transfer learning approach with freezing, we kept the convolutional layers of the pre-trained network frozen and only trained a new classifier on top of them using the same settings.

#### 2.3.3 AlternarAI model

In addition to the existing deep neural network architectures, we developed a custom CNN for binary classification of *Alternaria solani*, called AlternarAI. The AlternarAI model includes five convolutional layers, with filter size 3×3 and the number of channels ranging from 3 to 256, to capture different levels of detail in the input patches. To improve the training and generalization performance, a batch normalization layer is added after each convolutional layer, followed by a leaky rectified linear unit activation layer Xu et al. (2015).

The architecture also includes pooling layers. After the first four convolutional layers, max pooling is used with filter size 2×2, and after the fifth convolutional layer, average pooling with filter size 4×4.

The final layers of the architecture are two fully connected layers, which take the high-level features extracted by the convolutional layers and use them to classify the input data. The number of neurons in these layers is determined by the size of the input data after the last convolutional layer (3×3×256) and the number of classes to be classified, in our case 1 for binary classification. Right before each fully connected layer, a dropout layer with a probability of 0.3 is included, again to counteract overfitting and improve the generalization performance. The first fully connected layer is followed by a leaky rectified linear activation, the last one by a sigmoid activation to produce the binary output value.

The AlternarAI model is trained with a batch size of 128, with an initial learning rate of 0.001 and multistep approach, reducing the learning rate after 30 and 80 epochs by factor 0.1. Adam Kingma and Ba (2014) is used to update the parameters during training, with L2 regularization of 0.0001.

#### 2.3.4 Model evaluation

In this study, we employed a variety of evaluation metrics to quantitatively evaluate and compare the performance of our models. Specifically, we utilized accuracy, precision, recall, and F1 score metrics to assess the performance of our classification model. The formulas of these metrics are shown in Equations 2–5 respectively. When comparing various deep learning models, the evaluation metrics were calculated using a 5-fold cross-validation approach. For all other experiments, they were averaged over 10 runs and reported as mean ± standard deviation.


(2) $$\text{Precision}=\frac{TP}{TP+FP}$$

(3) $$\text{Recall}=\frac{TP}{TP+FN}$$

(4) $$\text{F}1\text{ score}=\frac{TP}{TP+0.5\times \left(FP+FN\right)}$$

(5) $$\text{Accuracy}=\frac{TP+TN}{TP+FP+TN+FN}$$

## 3 Results

We experimentally evaluate different aspects of the task of supervised Alternaria detection. In Subsection 3.1, we compare different model architectures to find a suitable model that provides a favorable trade-off between accuracy and computational cost. Taking the best-performing model based on this trade-off, we then evaluate its generalization capability by training and testing the model on data from different years in Subsection 3.2. In addition, in Subsection 3.3, we also evaluate the impact of the dataset size, class imbalance, and label quality on the final obtained accuracy. This provides useful guidelines to practitioners who are interested in collecting datasets for similar purposes. Finally, we show that the predictions of our model can be used to generate an overview heatmap of Alternaria infections that can then be used as a guide for the more precise application of plant protection products (Section 3.4).

### 3.1 Comparison of various deep learning models

We first compare our AlternarAI model with five other popular CNN architectures: ResNet50 He et al. (2015), VGG11 Simonyan and Zisserman (2015), InceptionV3 Szegedy et al. (2015), DenseNet161 Huang et al. (2016) and EfficientNetV2 Tan and Le (2021). For each model, we compare two strategies where either only the last layer was retrained (Shallow Transfer Learning: STL) or all layers were retrained (Deep Transfer Learning: DTL). The results are shown in **Tables 3** and **4**. These differ in the performance metric being used, respectively F1 score and accuracy, Precision and recall. We used 5-fold cross-validation to obtain all these results. The columns correspond to different subsets of the entire dataset being used for training and evaluation. These can either correspond to just a single year (2019, 2020, 2021, 2022) or a combination of multiple years (19-20-20 and 19-20-21-22). In all cases, the models are trained and evaluated on data from the same period. We found that finetuning all layers (DTL) typically outperforms the shallow transfer learning, presumably due to the low degree of similarity between ImageNet Fei-Fei et al. (2009) and the *Alternaria* dataset.

**Table 3:** Results of different architectures on various datasets using 5-fold cross-validation.

(A): F1 score						
Architecture	2019	2020	2021	2022	19-20-21	19-20-21-22
ResNet50 (STL)	0.73	0.77	0.65	0.77	0.80	0.80
VGG11 (STL)	0.86	0.69	0.62	0.73	0.77	0.75
InceptionV3 (STL)	0.84	0.60	0.54	0.62	0.72	0.69
DenseNet161 (STL)	0.88	0.76	0.62	0.79	0.81	0.82
EfficientNetV2 (STL)	0.83	0.67	0.64	0.64	0.76	0.76
ResNet50 (DTL)	0.91	0.88	0.90	0.91	0.91	0.91
VGG11 (DTL)	0.93	0.91	0.94	0.92	0.93	0.93
InceptionV3 (DTL)	0.93	0.90	0.87	0.90	0.92	0.91
DenseNet161 (DTL)	0.92	0.89	0.88	0.89	0.92	0.92
EfficientNetV2 (DTL)	0.93	0.90	0.89	0.92	0.92	0.92
AlternarAI	0.91	0.87	0.79	0.88	0.92	0.91
(B): Accuracy						
Architecture	2019	2020	2021	2022	19-20-21	19-20-21-22
ResNet50 (STL)	0.70	0.82	0.81	0.85	0.82	0.83
VGG11 (STL)	0.82	0.79	0.81	0.84	0.80	0.80
InceptionV3 (STL)	0.80	0.76	0.80	0.84	0.80	0.77
DenseNet161 (STL)	0.85	0.82	0.80	0.87	0.81	0.85
EfficientNetV2 (STL)	0.79	0.75	0.81	0.79	0.79	0.79
ResNet50 (DTL)	0.89	0.91	0.94	0.94	0.92	0.93
VGG11 (DTL)	0.92	0.93	0.97	0.95	0.93	0.94
InceptionV3 (DTL)	0.91	0.92	0.91	0.94	0.92	0.92
DenseNet161 (DTL)	0.91	0.92	0.93	0.93	0.92	0.93
EfficientNetV2 (DTL)	0.91	0.92	0.93	0.95	0.93	0.93
AlternarAI	0.89	0.90	0.89	0.92	0.92	0.93

STL, Shallow Transfer Learning; DTL, Deep Transfer Learning; 19-20-21, combined dataset of 2019, 2020 and 2021; 19-20-21-22, complete dataset of all years (2019, 2020, 2021, 2022).

The best score per year or combination of years is underlined in both subtables.

**Table 4:** Extra metrics of results of different architectures on various datasets using 5-fold cross-validation.

(A): Precision						
Architecture	2019	2020	2021	2022	19-20-21	19-20-21-22
ResNet50 (STL)	0.82	0.76	0.75	0.79	0.84	0.84
VGG11 (STL)	0.84	0.80	0.78	0.78	0.82	0.80
InceptionV3 (STL)	0.82	0.82	0.85	0.80	0.81	0.82
DenseNet161 (STL)	0.87	0.80	0.80	0.86	0.81	0.86
EfficientNetV2 (STL)	0.32	0.70	0.74	0.74	0.79	0.80
ResNet50 (DTL)	0.93	0.93	0.94	0.93	0.94	0.95
VGG11 (DTL)	0.94	0.92	0.94	0.93	0.92	0.93
InceptionV3 (DTL)	0.94	0.91	0.82	0.94	0.93	0.97
DenseNet161 (DTL)	0.93	0.90	0.91	0.90	0.92	0.93
EfficientNetV2 (DTL)	0.93	0.91	0.93	0.96	0.94	0.93
AlternarAI	0.92	0.89	0.91	0.91	0.93	0.92
(B): Recall						
Architecture	2019	2020	2021	2022	19-20-21	19-20-21-22
ResNet50 (STL)	0.71	0.78	0.59	0.76	0.79	0.78
VGG11 (STL)	0.89	0.61	0.51	0.69	0.73	0.73
InceptionV3 (STL)	0.86	0.48	0.40	0.51	0.66	0.61
DenseNet161 (STL)	0.89	0.73	0.55	0.74	0.82	0.78
EfficientNetV2 (STL)	0.82	0.64	0.58	0.56	0.73	0.73
ResNet50 (DTL)	0.89	0.85	0.86	0.89	0.88	0.87
VGG11 (DTL)	0.93	0.90	0.95	0.92	0.93	0.94
InceptionV3 (DTL)	0.92	0.89	0.93	0.87	0.90	0.85
DenseNet161 (DTL)	0.92	0.89	0.87	0.90	0.92	0.91
EfficientNetV2 (DTL)	0.93	0.88	0.85	0.88	0.90	0.91
AlternarAI	0.90	0.85	0.71	0.85	0.91	0.91

STL, Shallow Transfer Learning; DTL, Deep Transfer Learning; 19-20-21, combined dataset of 2019, 2020 and 2021; 19-20-21-22, complete dataset of all years (2019, 2020, 2021, 2022).

The best score per year or combination of years is underlined in both subtables.

As shown in **Table 3**, the F1 scores of the deep transfer learned models and AlternarAI fluctuate among the different datasets and models. However, similar values of F1 score were observed for the different deep transfer learned models. When using datasets combining different years (19-20-21 and 19-20-21-22), the F1 scores vary between 0.91 and 0.93 when performing 5-fold cross-validation. The F1 scores of the AlternarAI model are 0.92 and 0.91, for 19-20-21 and 19-20-21-22 respectively, and thus comparable to the F1 scores of the other deep transfer learned models. The results in terms of precision and recall are also very similar for the complete dataset, as well as for the combination of 2019, 2020 and 2021, which will be used as a training set in further experiments. For the individual years, AlternarAI scores were slightly lower than ResNet50 (DTL), EfficentNetV2 (DTL), InceptionV3 (DTL) and VGG11 (DTL). Furthermore, it can be seen that VGG11 consistently demonstrates slightly better results than the other models in terms of F1 score, accuracy, and recall.

While the obtained F1 score is of course important for the practical applicability of the model, it is not the only concern, most models obtain a similar performance hence other elements such as the inference time or the memory required to evaluate the model might be crucial to select the best model. **Table 5** shows the difference in trainable parameters and inference time between all models. Most state-of-the-art architectures have a very large number of trainable parameters, whereas the AlternarAI model has at least fifteen times fewer parameters. In addition, the AlternarAI had a lower inference time (1.49 ms) compared to the state-of-the-art architectures, particularly to DenseNet161 (DTL) and EfficentNetV2 (DTL), models with only slightly higher F1 scores than AlternarAI. The difference in inference time with VGG11 is limited, however, VGG11 has more than 80 times the total number of parameters compared to the AlternarAI model, making it less suitable for memory-constrained devices. Thanks to its excellent trade-off between accuracy, inference time and memory consumption, the AlternarAI model was selected for all subsequent experiments.

**Table 5:** Amount of parameters in the model, mean inference time (ms) of 5 folds per image patch of 256×256 pixels and total processing time on field set, based on an average of 528 images per flight.

Architecture	Parameters (Trainable subset)	Mean inference time (ms)	Total processing time on field set (min)
ResNet50 (STL)	23.77×10^6^ (0.26×10^6^)	9.35 ± 1.28	51.01
VGG11 (STL)	128.77×10^6^ (4.10×10^3^)	1.79 ± 0.10	9.77
InceptionV3 (STL)	24.35×10^6^ (2.82×10^3^)	18.10 ± 2.15	98.75
DenseNet161 (STL)	26.75×10^6^ (0.28×10^6^)	28.89 ± 1.50	157.62
EfficientNetV2 (STL)	20.34×10^6^ (0.16×10^6^)	22.12 ± 0.61	120.69
ResNet50 (DTL)	23.77×10^6^	9.34 ± 0.36	50.96
VGG11 (DTL)	128.77×10^6^	1.91 ± 0.44	10.42
InceptionV3 (DTL)	24.35×10^6^	18.16 ± 0.98	99.08
DenseNet161 (DTL)	26.75×10^6^	28.86 ± 0.53	157.46
EfficientNetV2 (DTL)	20.34×10^6^	22.29 ± 0.28	121.61
AlternarAI	1.57×10^6^	1.49 ± 0.09	8.13

### 3.2 Generalization over different years

**Figure 3:**
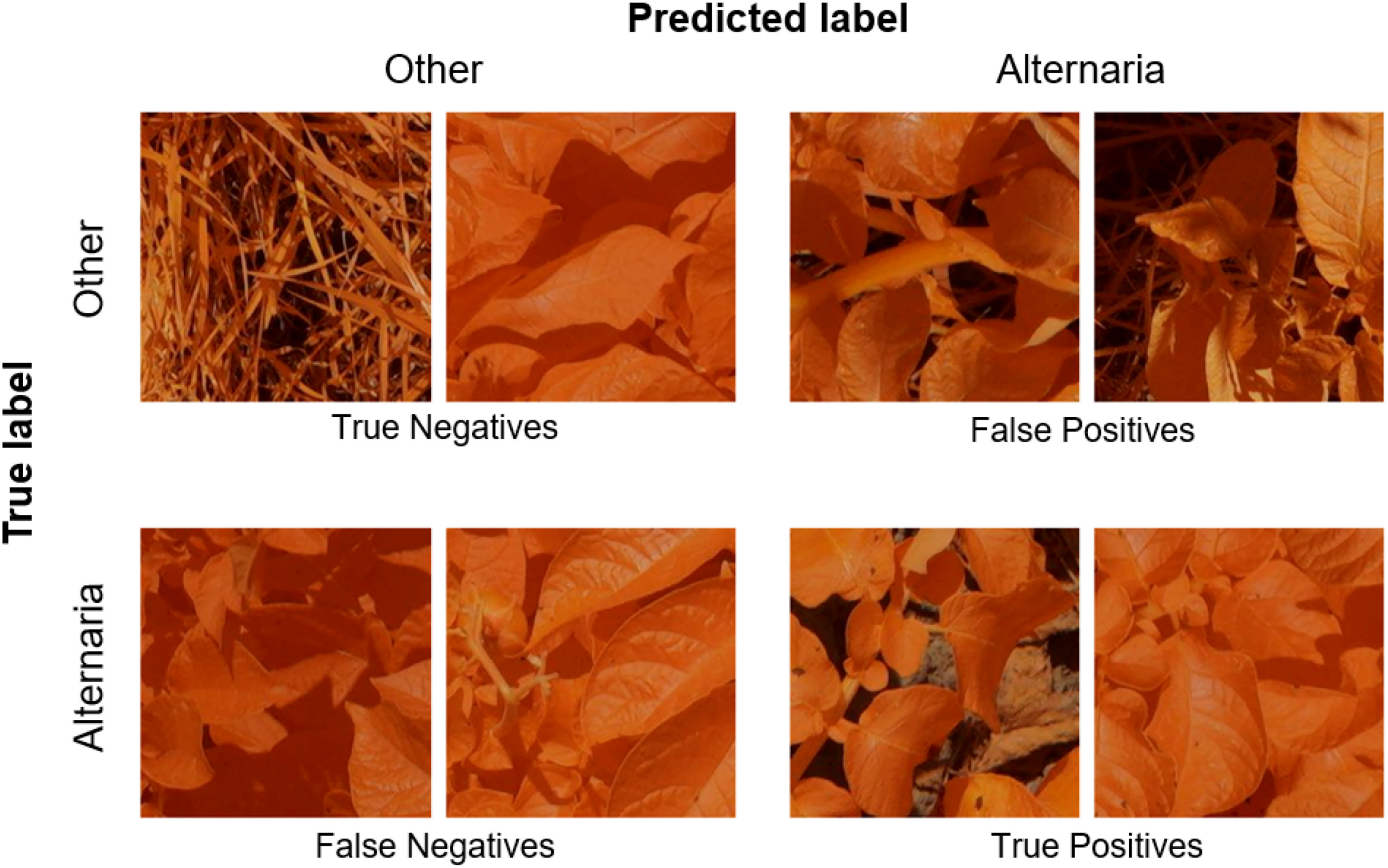
Example patches of True Negatives (top left), False Negatives (bottom left), False Positives (top right) and True positives (bottom right).

In the previous Section, we evaluated the model on data from a single growing season, as is common in existing work. In this Section, we however want to evaluate how well the model generalizes to data from unseen years. A model that is robust against the differences in environmental factors between different years is much easier to use in practice. To evaluate this, we trained the AlternarAI model on subsets of the data of a single year and tested it on the completely independent subsets of all other years. We report both the accuracy and F1 score, averaged over 10 runs in **Table 6**. The F1 score varies between 0.65 and 0.83 on the individual years, but reaches 0.89 when trained on a combined dataset of 2019, 2020, and 2021 and tested on the set of 2022. For the remainder of the experiments, we use this best-performing model. **Table 7** is the normalized confusion matrix for this model, showing that indeed, the model is making accurate predictions. For completeness, we also include some qualitative results in **Figure 3** where we show two example patches of each category (i.e., true negatives, false negatives, false positives and true positives).

**Table 6:** Results of testing a trained model on a completely independent dataset (averaged over 10 runs).

(A): Accuracy						
		Tested on…				
		2019	2020	2021	2022	19-20-21
Trained on…	2019	/	0.77±.0.03	0.73±0.05	0.87±0.02	/
	2020	0.80±0.02	/	0.88±0.12	0.84±0.03	/
	2021	0.79±0.01	0.81±0.01	/	0.84±0.02	/
	2022	0.91±0.03	0.79±0.08	0.69±0.08	/	0.78±0.05
	19-20-21	/	/	/	**0.93±0.01**	/
(B): F1 score						
		Tested on…				
		2019	2020	2021	2022	19-20-21
Trained on…	2019	/	0.75±0.02	0.68±0.04	0.82±0.02	/
	2020	0.83±0.01	/	0.82±0.02	0.74±0.05	/
	2021	0.81±0.01	0.70±0.03	/	0.71±0.04	/
	2022	0.83±0.03	0.76±0.08	0.65±0.06	/	0.77±0.05
	19-20-21	/	/	/	**0.89±0.01**	/

The bold values show the result of the model trained on the data of 2019, 2020 and 2021 together, tested on the independent set of 2022.

**Table 7:** Resulting normalized confusion matrix, trained on 2019, 2020 and 2021 data, tested on 2022 data, averaged over 10 runs.

		Predicted	
		Other	Alternaria
True	Other	0.93	0.07
	Alternaria	0.09	0.91

### 3.3 Impact of dataset characteristics

As is common in machine learning applications, the performance of the model heavily depends on the training data being used. In this section, we investigate the influence of three main properties of a dataset: (i) the size, (ii) the balance between both classes and (iii) the correctness of the labels. We hope that these findings will serve as a guideline for practitioners who are interested in collecting their dataset for a similar task.

**Figure 4:**
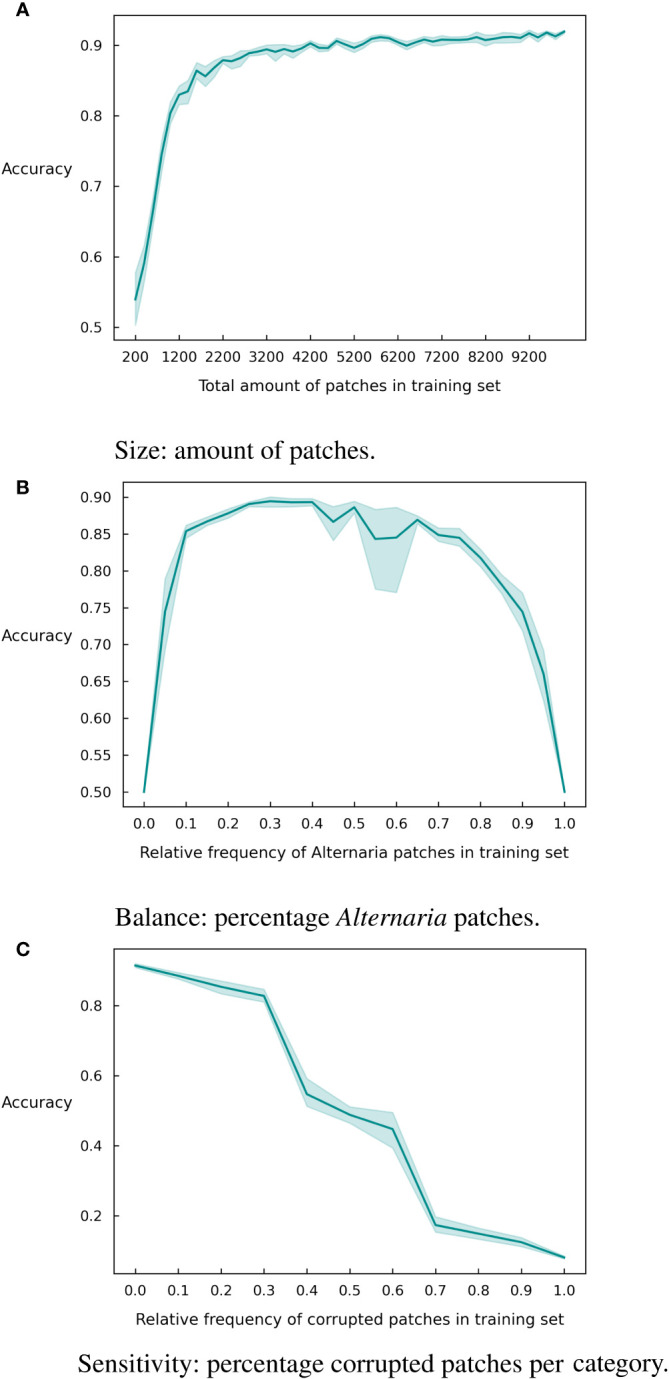
Graphical representation of different dataset characteristics and their effect on the accuracy with 95% confidence intervals over 10 runs. **(A)** Size. **(B)** Balance. **(C)** Sensitivity.

Since deep learning is known for its demand for large datasets, we first evaluated the impact of the amount of patches in the training set. As can be observed in **Figure 4A**, the model accuracy increases with an increase in the number of patches in the training set. However, a stagnation of the curve occurs, at about 4200 patches where the model obtains an accuracy of 0.90. Slightly lower than the final accuracy after 10,000 patches (0.92). While this difference in accuracy is most likely not critical, it can significantly reduce the data labeling cost.

In addition to the size of the training set, the imbalance between the classes might also be significant for the accuracy of the model. To investigate this, we created imbalanced versions of the training dataset and retrained the model. We varied the number of *Alternaria* patches between 0% and 100% of the total training dataset and show the resulting accuracy in **Figure 4B**. The class imbalance has a particularly negative effect when it is below 20% or above 75%. So, an extremely skewed dataset will influence the results, but there is margin for a reasonably large imbalance in the set.

Finally, we investigate the robustness of the model to the quality of the labels. Data labeling is largely a human effort and is thus prone to human errors. In this paper, we addressed this by having each sample labeled multiple times by independent labelers. This results in high quality labels but is of course very expensive. If the model is robust against a significant number of labeling errors, it might suffice to have a single label for each sample, resulting in a much more inexpensive labeling campaign. To investigate the robustness, we created corrupted subsets of the training data where we inversed the label. **Figure 4C** shows the accuracy of the model for different corruption rates. As expected, the performance of the model degrades significantly for increasing corruption rates but even at a rate of 30% mislabeled inputs, the model is still able to achieve an accuracy of 80%.

### 3.4 Field-level detection

**Figure 5:**
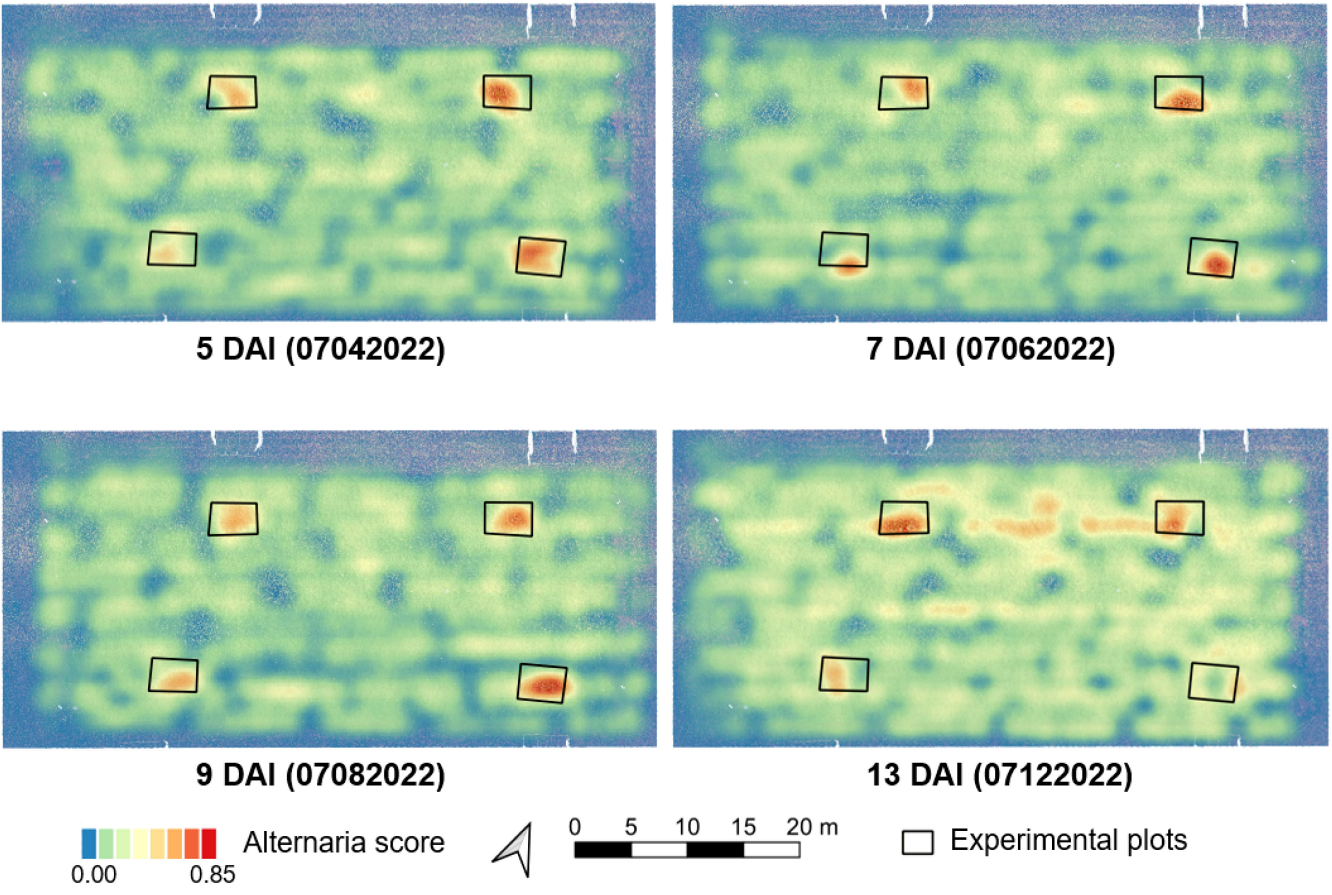
Overview maps of the predicted Alternaria score at different days after inoculation (DAI) in 2022. These predictions were made by the AlternarAI model trained on data from previous years (2019, 2020 and 2021). The black rectangles indicate zones that were infected manually. Regions with a red color indicate higher predicted Alternaria rates.

In all previous sections, we limited ourselves to small individual patches. In this section, we combine all individual predictions into a large-scale overview of the entire field. This allows us to quickly identify regions of the field that are potentially infected and that might warrant further inspection or treatment. **Figure 5** shows the results for four different days after inoculation (DAI). For each individual patch in the image, the model predicts a score between 0 and 1. We then average out these predictions of nearby patches to obtain an overview heatmap. Regions that are indicated in red have a higher number of patches that are predicted to contain Alternaria. We can clearly see the four infected regions on each image, annotated with a black rectangle. For these patches, the model accurately predicts a much higher Alternaria rate, even only five days after inoculation. Interestingly, the model also detected Alternaria outside these zones on data of the last day. Upon further inspection, we found that these plants were indeed infected.

**Figure 6:**
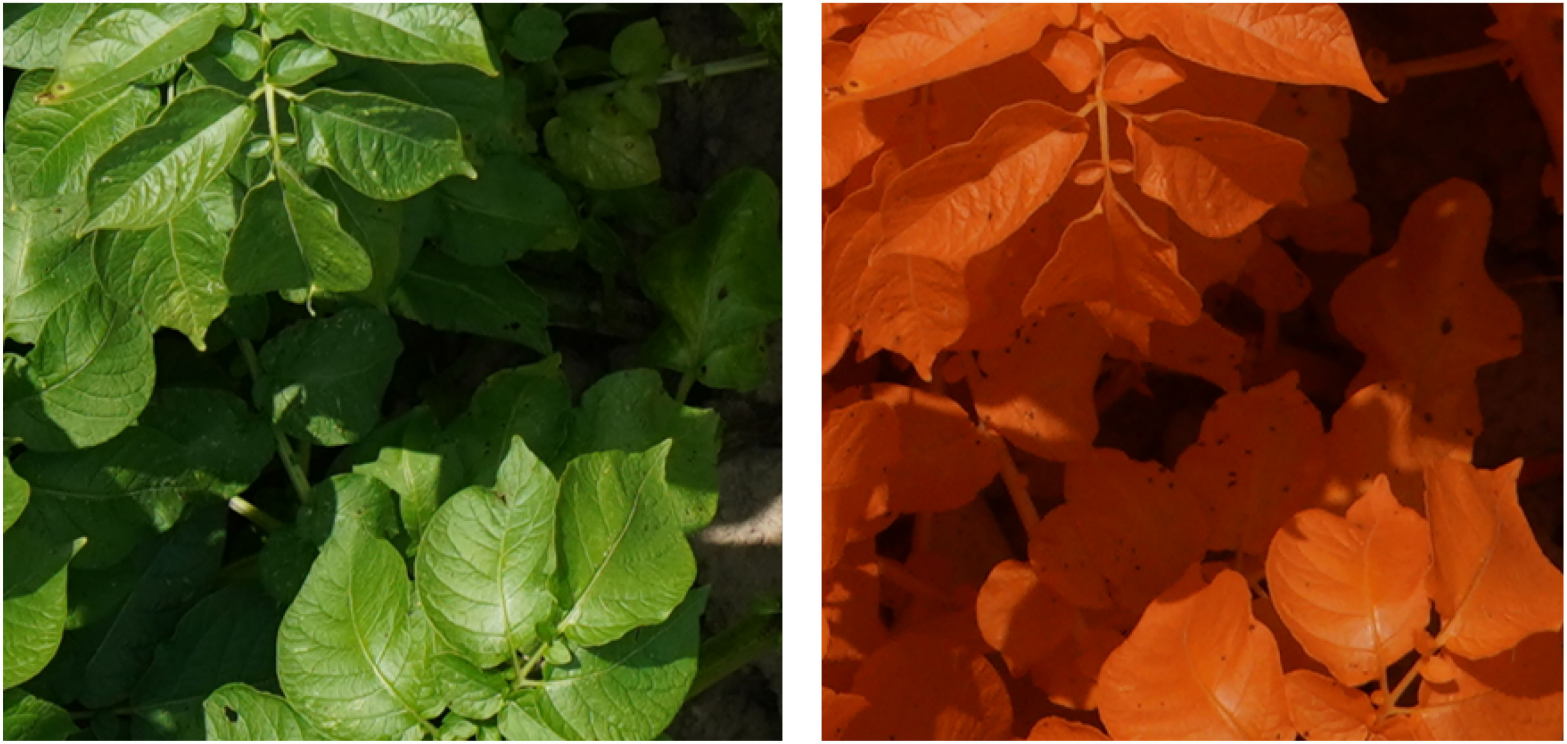
Close-up from both RGB (left) and Modified RGB (right) image in the upper rightmost *Alternaria* plot of the 4th of July 2022 (5 DAI).

As can be seen in **Figure 5**, the model is able to distinguish the images captured above *Alternaria* plots from the other regions in and around the field, even five days after inoculation. **Figure 6** shows a small part of an image taken at 07/04/2022 (5 DAI) in the upper rightmost *Alternaria* plot, both the RGB version as well as the Modified RGB version. The model classified this image as *Alternaria*, although it is almost invisible to see the *Alternaria* lesions in the normal RGB image.

## 4 Discussion

In recent years, interest in the use of deep learning for disease detection in the precision agriculture context increased dramatically. Unfortunately, most of these studies are limited in the variability of the data used. They typically train and test their models on data from the same field recorded during the same year. Hence, they must be considered as a proof of concept rather than as operational methods directly applicable in practice. After all, robust supervised models require a sufficiently large, diverse, representative and labeled dataset Lu et al. (2021). Only when a model performs well on a completely independent dataset, it can be considered robust. Image data in field conditions can differ significantly due to variations in plants (e.g., size, shape, cultivar), variations in disease progress, as well as variations in measurement conditions (e.g., illumination and weather conditions).

This study aims to provide a more realistic exploration of using deep learning models in the context of early blight detection in potato fields. We used UAVs to collect a very large dataset, covering a wide variety of measurement conditions and spanning multiple years.

Our large, labeled dataset of 14,057 image patches allows us to quantitatively compare different state-of-the-art neural network models. For this experiment, we combined data from all four years and evaluated the performance of different models using 5-fold cross validation. Similar accuracies (0.92-0.93) and F1 scores (0.91-0.92) were observed for all models. We then developed our own AlternarAI architecture, based on the best performing VGG11 model. Our model obtains a similar performance, yet is much smaller compared to the other models (**Table 5**), reducing the computational cost and training time. In addition, we also compare the inference time of the different models since this is an important constraint in practical applications Johnson et al. (2021). We found that our AlternarAI model is significantly faster than most baseline approaches. Only VGG11 has a similar inference time. While the processing time for a single patch is relatively low (1-30 ms), the overall compute time to process all data from an entire flight quickly adds up. It would for example take 43 minutes longer to process one flight using ResNet50 than using AlternarAI. We found that our AlternarAI model provides the best trade-off between accuracy, computational cost and memory consumption.

Our large dataset, spanning multiple years also allows us to investigate other properties of these deep learning models that are typically not addressed in similar works. We analyzed how well models are able to generalize to data of other years and other cultivars. While we did obtain fluctuating F1 scores for different years, as presented in **Table 6**, we also found that a model trained on combined data of 2019, 2020 and 2021 performed very well on data from 2022. We attribute this to the wide variety of different cultivars, weather and capturing conditions present in the training data.

In a next series of experiments, we assessed the influence of three different dataset characteristics on the final accuracy of the model, as an extension of what we described in a preliminary study [Wieme et al. (2022)]. We first measured the impact of the amount of training data. **Figure 4A** clearly shows that up to a certain threshold point, additional data directly improves accuracy. The threshold amount here is slightly higher than observed in our previous work [Wieme et al. (2022)]. This is possibly because of the fact that in previous work the test and training set were both selected from the data of 2019 and 2020, and thus had a more equal distribution, whereas in this paper a total new test set of 2022 is used.

A second aspect to take into account when training supervised models is the balance between different classes. In **Figure 4B**, the same trend as observed in our previous work Wieme et al. (2022) with data from 2019 and 2020 can be seen: a perfect balance in the training dataset is not required. Here, we see that ratios between 20% and 70% of *Alternaria* patches deliver comparable results in overall accuracy. In previous experiments, we saw that models trained and tested on the datasets of 2021 and 2022 experience more difficulties than with 2019 and 2020 sets. Besides the fact that these two are the smallest, the balance is 70-30% in 2021 and 67-32% in 2022, which already appears to be on the edge of what is acceptable.

Finally, we analyzed the sensitivity of the model to incorrect labeling (3c). In agreement with previous findings [Wieme et al. (2022)], the accuracy initially decreases steadily as the amount of wrongly labeled data points increases. Once past 30% corrupt labels, a steeper drop can be noticed. After 50%, we see a symmetric pattern in the reverse direction, since the model receives more incorrect than correct information, leading the model to learn to make exactly opposite predictions.

These results about dataset characteristics are in accordance with what Johnson et al. (2021) discussed: the foundation of a successful model is the quality and quantity of the training data. Collecting a large, high-quality dataset is a time- and cost-consuming process, especially when experts must apply manual labeling. Thus, our results provide worthwhile advice for acquiring similar datasets, which is to collect a sufficiently large dataset, with a ratio of at least 25% between both classes and with careful focus on labeling.

We also performed qualitative experiments where we further evaluated the performance of the trained model (trained on the patches of 2019, 2020 and 2021) on the full images of 2022. By using this varied dataset rather than a single time point dataset, the model became more robust. This is illustrated in **Figure 5**, where images in/around the infected plots are clearly classified as *Alternaria*, in contrast with images in the rest of the field, on different measurement days of an independent set. These maps can form the baseline for task maps for variable spraying. The whole inference workflow is shown in the right half of **Figure 2**.

**Figure 7:**
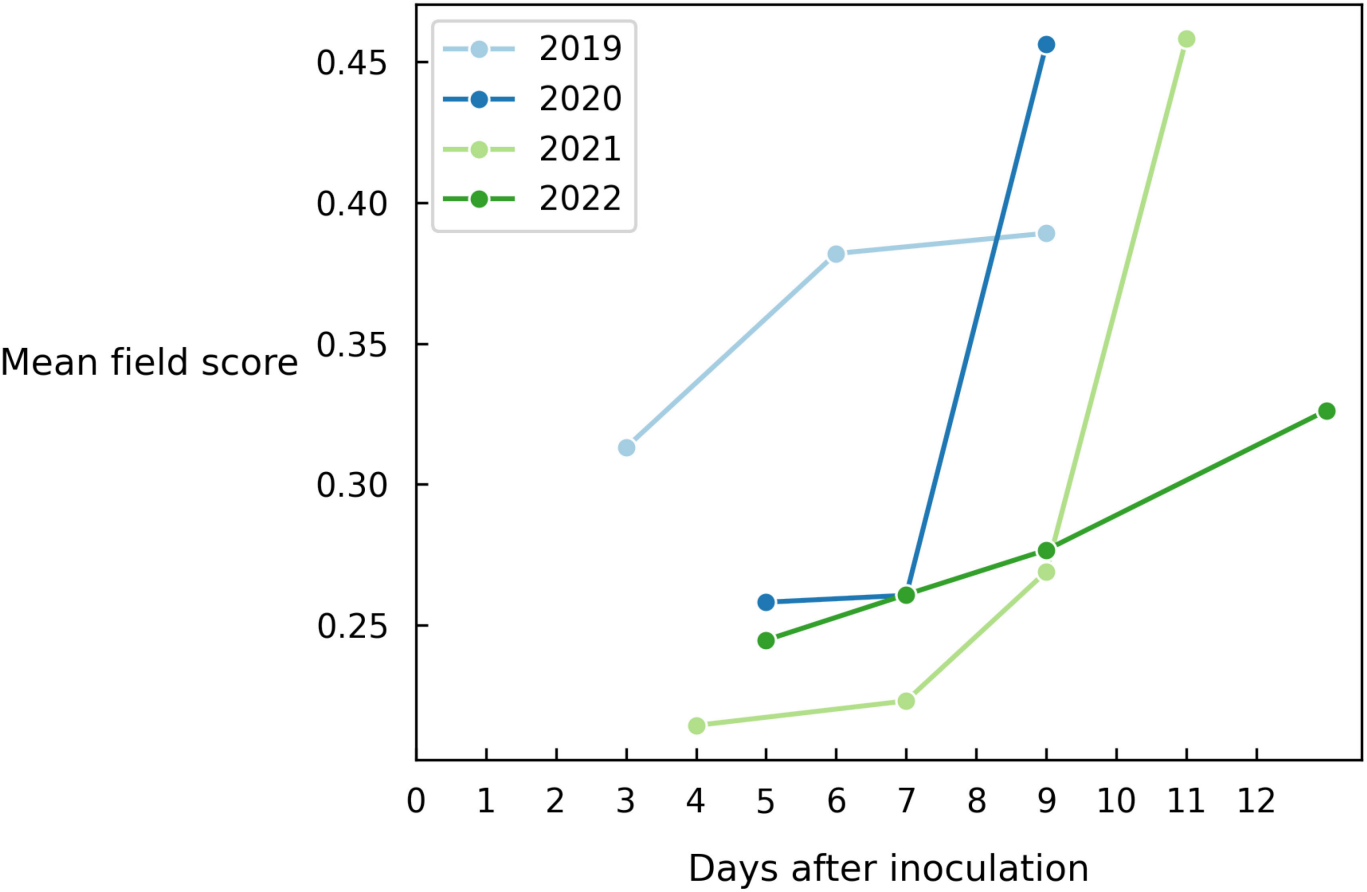
Evolution of the mean field disease score for different years on all images of each measurement day.

As a final experiment, we average out all predictions of the entire field to obtain a single value indicating to what level Alternaria is present in the field (**Figure 7**). This metric also depends on how the data was collected in that year, which means that the absolute score across years should not be compared. However, we clearly observe a similar disease progression each year.

## 5 Conclusion and future work

The challenges of creating a model applicable in practice start with the collection of a representative and extensive data set. Therefore, we collected a very large dataset of in-field modified RGB, ultra-high-resolution UAV-images of both healthy and diseased potato plants at different times in the disease progress during multiple growing seasons. Based on this dataset, we developed a robust convolutional neural network (CNN), AlternarAI, to perform a binary classification for the detection of *Alternaria solani*. Four state-of-the-art CNNs were trained for the binary classification of early blight. ResNet50, InceptionV3 and DenseNet161 were able to distinguish between diseased and healthy images with sufficient accuracy. However, the results also demonstrated that the AlternarAI model achieves similar accuracy and becomes the best performing model in conjunction with lower inference time. Both quantitative and qualitative experiments showed that the model is able to generalize well on independent test sets of other growing seasons. Furthermore, we assessed the importance of three dataset characteristics. The experiments showed that the amount and quality of labeled patches have a direct impact on the accuracy, and a reasonable balance, although not a perfect one, is preferred. In conclusion, the addition of variability in data results in more robust disease detection, which is desired for in-field application. The complete workflow of both training and testing is visually represented in **Figure 2**. Therefore, this method can be perceived as a basis for decision support systems, as an aid for farmers to monitor their field, and as input for a variable spraying system to lower the use of crop protection products. In future work, we will focus on reducing the labeling effort using active learning or semi-supervised models.

## Data availability statement

The dataset of 2019 and 2022 presented in this study is publicly available at 10.5281/zenodo.10727413. If you use this dataset, please refer to this journal article.

## Author contributions

Conceptualization, JW, SL, and WM; Data Collection, JW, SC, and WM; Writing-Original Draft Preparation, JW. Writing-Review and Editing, SC, SL, JP, and WM; Supervision, SC, SL, JP, and WM. JV: Writing – review & editing, Data curation, Methodology, Supervision. All authors contributed to the article and approved the submitted version.
